# In this month’s *Bulletin*

**DOI:** 10.2471/BLT.15.000915

**Published:** 2015-09-01

In the editorial section, Ahmad Reza Hosseinpoor et al. (591) stress the importance of monitoring health inequality in the post-2015 agenda. James Campbell et al. (590) put the cost–effectiveness study done by Barbara McPake et al. (631–640) into the global context of using community-based practitioners to expand access to health care. 

In the news section, Apiradee Treerutkuarkul and Karl Gruber report on efforts to improve oral health in low- and middle-income countries (594–595). Sheila Tlou tells Fiona Fleck how HIV prevention campaigns have evolved in Botswana since the 1980s (596–597).

## Argentina

### Improving the nation’s diet

Adolfo Rubinstein et al. (614–622) estimate the health and economic impacts of a national policy to eliminate trans fats. 

## Central African Republic, Democratic Republic of the Congo, Guinea, India, Kenya, Lesotho, Malawi, Mozambique and South Africa

### Tracking inaccurate results 

Emmanuel Fajardo et al. (623–630) examine invalid test results generated by a CD4+ analyser from sites in nine countries. 

## China

### Disclosing medical errors

Lei Gao et al. (661–662) consider cultural influences on informed consent and disclosure practices. 

## Cyprus

### Making medicines affordable

Olivier J Wouters & Panos G Kanavos (606–613) consider potential policy solutions for high costs in the pharmaceutical sector. 

## Ethiopia, Indonesia and Kenya

### Sourcing health care from the community

Barbara McPake et al. (631–640) analyse the cost–effectiveness of community-based practitioner programmes. 

## Japan

### Risk perception and mental health

Yuriko Suzuki et al. (598–605) investigate the perception of radiation risks and psychological distress among evacuees of the Fukushima Daiichi nuclear power plant accident.

## Sri Lanka

### Lives lost to more roads

Samath D Dharmaratne et al. (641–648) analyse trends over 75 years in road traffic crashes, injuries and deaths. 

## Global 

### Collecting data in emergency settings

Thidar Pyone et al. (649–660) review the tools available for rapid assessments of maternal and child health. 

### Understanding complex problems

David Bishai et al. (663–664) explain why communities affected by a public health problem should describe and address it. 

